# Association of Race and Ethnicity With Prostate Cancer–Specific Mortality in Canada

**DOI:** 10.1001/jamanetworkopen.2021.36364

**Published:** 2021-12-21

**Authors:** Noah Stern, Tina Luu Ly, Blayne Welk, Joseph Chin, Dale Ballucci, Michael Haan, Nicholas Power

**Affiliations:** 1 Division of Urology, Department of Surgery, Western University, London, Ontario, Canada; 2 Department of Sociology, Western University, London, Ontario, Canada

## Abstract

This cohort study uses data from the Canadian Census Health and Environment Cohorts to assess the association of race and ethnicity with prostate cancer–specific mortality among men in Canada.

## Introduction

Despite extensive research, age, family history, race and ethnicity, and certain genetic sequence variants remain the only currently well-established risk factors for prostate cancer. Black men are purported to have an increased risk of early and aggressive prostate cancer. This higher risk classification for Black men has resulted in recommendations for earlier prostate cancer screening and evidence of distinctive treatment patterns.^1,2^ However, the underlying data for these recommendations lack high-quality evidence, rendering the validity of these conclusions uncertain. Data from the Canadian health care system are well suited for studying the association of race and ethnicity and prostate cancer mortality, owing to Canada’s diverse population and universal health care model.

## Methods

This cohort study was approved by the Western University Institutional Research Ethics Board. Statistics Canada datasets were used for the research; these datasets incorporate implied consent from Canadian citizens and are subject to stringent reporting and confidentiality guidelines in accordance with the Statistics Act. The study followed the Strengthening the Reporting of Observational Studies in Epidemiology (STROBE) reporting guideline.

Data from the Statistics Canada Canadian Census Health and Environment Cohorts were used.^3^ The mandatory 1991 long-form census (administered to 20% of households) was linked with Canadian health care, tax, and mortality databases (including the Canadian Vital Statistics Death Database, the Canadian Cancer Registry, and the historical postal code file from tax records) to investigate the association of race and ethnicity with prostate cancer mortality in Canada. Individuals who completed the census chose from existing options to categorize their race and ethnicity, with an optional write-in response to describe their ancestry. The categories included in this study were Black, East Asian, South Asian, Southeast Asian or Filipino, West Asian or Arab, and a combined group comprising those who identified as White; White and Latin American, Arab, or West Indian; Latin American, Arab, or West Asian with a write-in response indicating European ancestry; or Aboriginal. All men diagnosed with prostate cancer between 1992 and 2010 were included. A diagnosis of or death attributable to prostate cancer was established using *International Classification of Diseases, Ninth Revision, Clinical Modification* code 185 or *International Classification of Diseases, Tenth Revision, Clinical Modification* code C61, respectively (with 95.9% sensitivity and 96.2% specificity).^4^ Cox proportional hazards models were used to predict the association of race and ethnicity as self-reported in the 1991 census and prostate cancer mortality after controlling for covariates including immigration status, age, education, and Canadian region of residence. Statistical analyses were performed with SAS version 9.4 (SAS Institute Inc). All counts are weighted and rounded, and percentages are based on weighted, rounded counts.

## Results

This study included 51 530 men who received a prostate cancer diagnosis between 1992 and 2010, with a median time to event of 6.5 (IQR, 2.8-9.8) years (Table 1). The mean (SD) age was 59 (12) years. Of these 51 530 men, 1080 (2.1%) indicated on census forms that they were Black; 725 (1.4%), East Asian; 510 (1.0%), South Asian; 210 (0.4%), Southeast Asian or Filipino; and 325 (0.6%), West Asian or Arab. A total of 48 680 men (94.5%) reported that they were White; White and Latin American, Arab, or West Indian; Latin American, Arab, or West Asian with a write-in response indicating European ancestry; or Aboriginal. In total, 29 705 men died, and 7925 of these deaths were attributable to prostate cancer. There was a lower risk of prostate cancer–specific mortality among South Asian men (hazard ratio [HR], 0.53 [95% CI, 0.36-0.76]) and East Asian men (HR, 0.62 [95% CI, 0.49-0.79]) compared with the reference group that included White; White and Latin American, Arab, or West Indian; Latin American, Arab, or West Asian with a write-in response indicating European ancestry; or Aboriginal individuals. No increased risk of prostate cancer–specific mortality was observed among Black men (HR, 0.83 [95% CI, 0.67-1.02]) (Table 2).

**Table 1.** Baseline Characteristics of Men in the 1991 CanCHEC With Prostate Cancer Who Died of Any Cause or of Prostate Cancer Between 1992 and 2010

Covariate	No. of men diagnosed with prostate cancer (N = 51 530)	No. of deaths			
		From any cause (n = 21 785)	*P* value	From prostate cancer (n = 7925)	*P* value
Racial and ethnic minority group^a^					
Black	1080	235	<.001	95	<.001
East Asian	725	215		70	
South Asian	510	125		30	
Southeast Asian, Filipino	210	55		15	
West Asian, Arab	325	110		35	
White and selected other races and ethnicities^b^	48 680	21 045		7675	
Immigration status					
Immigrant	12 805	5095	<.001	1795	<.001
Not an immigrant	38 725	16 695		6130	
Age, y					
25-34	755	25	<.001	20	<.001
35-44	5545	325		185	
45-54	12 635	1740		750	
55-64	16 185	6105		2225	
≥65	16 415	13 590		4750	
Education					
No high school	22 440	12 235	<.001	4420	<.001
High school	16 835	6210		2270	
Postsecondary	4905	1495		575	
University degree	7350	1845		660	

Abbreviation: CanCHEC, Statistics Canada Canadian Census Health and Environment Cohort.

^a^ As self-reported on the 1991 long-form census. Categorical grouping was required to ensure that the sample size adhered to Statistics Canada Research Data Centre confidentiality and reporting guidelines. To be included, men had to be aged 25 years or older at the time of the census. All numbers were weighted and rounded; therefore, subcategory numbers for a given category may not sum to the total for that category.

^b^ This group included White; White and Latin American, Arab, or West Indian; Latin American, Arab, or West Asian with a write-in response indicating European ancestry; or Aboriginal individuals.

**Table 2.** Multivariate Analysis of All-Cause and Prostate Cancer–Specific Mortality Among Canadian Men in the 1991 CanCHEC Diagnosed With Prostate Cancer Between 1992 and 2010

Covariate	All-cause mortality		Prostate cancer–specific mortality	
	HR (95% CI)	*P* value	HR (95% CI)	*P* value
Racial and ethnic minority group^a^				
White and selected other races and ethnicities^b^	1 [Reference]		1 [Reference]	
Black	0.76 (0.67-0.87)	<.001	0.83 (0.67-1.02)	.07
East Asian	0.65 (0.57-0.74)	<.001	0.62 (0.49-0.79)	<.001
South Asian	0.83 (0.69-0.99)	.04	0.53 (0.36-0.76)	<.001
Southeast Asian, Filipino	0.82 (0.63-1.07)	.14	0.68 (0.42-1.11)	.12
West Asian, Arab	1.14 (0.94-1.37)	.18	1.03 (0.74-1.43)	.88
Immigration status				
Not an immigrant	1 [Reference]		1 [Reference]	
Immigrant	0.89 (0.85-0.91)	<.001	0.87 (0.83-0.92)	<.001
Education				
No high school	1 [Reference]		1 [Reference]	
High school	0.82 (0.8-0.85)	<.001	0.83 (0.79-0.87)	<.001
Postsecondary	0.74 (0.70-0.78)	<.001	0.78 (0.71-0.85)	<.001
University degree	0.61 (0.58-0.64)	<.001	0.60 (0.55-0.65)	<.001

Abbreviations: CanCHEC, Statistics Canada Canadian Census Health and Environment Cohort; HR, hazard ratio.

^a^ As self-reported on the 1991 long-form census. Categorical grouping was required to ensure that the sample size adhered to Statistics Canada Research Data Centre confidentiality and reporting guidelines.

^b^ This group included White; White and Latin American, Arab, or West Indian; Latin American, Arab, or West Asian with a write-in response indicating European ancestry; or Aboriginal individuals.

## Discussion

To our knowledge, this study is the largest to date to assess the association of race and ethnicity and prostate cancer–specific mortality in Canadian men. Similar to previous high-quality research studies, South Asian and East Asian men in this study had lower rates of prostate cancer–specific mortality.^5^ Contrary to guideline statements, Black men in this study did not have an increased rate of prostate cancer–specific mortality.^1^

Black men have traditionally been classified as being at high risk for prostate cancer. However, it is unclear whether the increased risk of mortality is truly attributable to prostate cancer or whether there are social and societal barriers confounding poorer outcomes. The results of our study are consistent with recent pooled analyses of administrative data sets in which adjusting for nonbiological differences, including socioeconomic status and health care access, nearly eliminated the increased risk of prostate cancer–specific mortality observed among Black men in the US.^6^

Our results contradict earlier studies suggesting that Black men may have a biologically distinct form of aggressive prostate cancer. These findings may have implications for future prostate cancer screening and treatment guidelines.

We accept the limitations to our study and data set, although we believe them to be smaller than in previously published works. These limitations include the limited access to pathology data and the retrospective and administrative nature of these data sets, which limits the ability to stratify beyond broad ethnic groups and to control for family history or treatment modality. In addition, variable prostate-specific antigen screening rates and more aggressive treatment may affect observed survival.

Overall, this cohort study highlights the importance of addressing socioeconomic barriers to health care and, just as importantly, emphasizes the need for caution when drawing conclusions from observational studies.

## References

[1] Mottet N, Bellmunt J, Bolla M, . EAU-ESTRO-SIOG Guidelines on Prostate Cancer. Part 1: screening, diagnosis, and local treatment with curative intent. Eur Urol. 2017;71(4):618-629. doi:10.1016/j.eururo.2016.08.003 27568654

[2] Chornokur G, Dalton K, Borysova ME, Kumar NB. Disparities at presentation, diagnosis, treatment, and survival in African American men, affected by prostate cancer. Prostate. 2011;71(9):985-997. doi:10.1002/pros.21314 21541975 PMC3083484

[3] Tjepkema M, Christidis T, Bushnik T, Pinault L. Cohort profile: the Canadian Census Health and Environment Cohorts (CanCHECs). Health Rep. 2019;30(12):18-26. doi:10.25318/82-003-X201901200003-ENG 31851369

[4] Godtman R, Holmberg E, Stranne J, Hugosson J. High accuracy of Swedish death certificates in men participating in screening for prostate cancer: a comparative study of official death certificates with a cause of death committee using a standardized algorithm. Scand J Urol Nephrol. 2011;45(4):226-232. doi:10.3109/00365599.2011.559950 21463227

[5] Miller BA, Chu KC, Hankey BF, Ries LAG. Cancer incidence and mortality patterns among specific Asian and Pacific Islander populations in the U.S. Cancer Causes Control. 2008;19(3):227-256. doi:10.1007/s10552-007-9088-3 18066673 PMC2268721

[6] Dess RT, Hartman HE, Mahal BA, . Association of black race with prostate cancer-specific and other-cause mortality. JAMA Oncol. 2019;5(7):975-983. doi:10.1001/jamaoncol.2019.0826 31120534 PMC6547116

